# Oil Extraction and Evaluation from Yellow Horn Using a Microwave-Assisted Aqueous Saline Process

**DOI:** 10.3390/molecules24142598

**Published:** 2019-07-17

**Authors:** Yulong Huang, Zhenxiong Yin, Jie Guo, Fengxia Wang, Ji Zhang

**Affiliations:** 1 Gansu Innovation Center of Fruit and Vegetable Storage and Processing, Agricultural Product Storage and Processing Institute, Gansu Academy of Agricultural Sciences, Lanzhou 730070, China; 2 Bioactive Products Engineering Research Center for Gansu Distinctive Plants, College of Life Science, Northwest Normal University, Lanzhou 730070, China

**Keywords:** yellow horn, aqueous salt process, Box–Behnken design, antioxidant activities, fatty acids

## Abstract

This study investigates an aqueous salt process (ASP) combined with microwave-assisted extraction (MAE) for the seed oil extraction from yellow horn (*Xanthoceras sorbifolium* Bunge). The NaCl concentration in the oil extraction process affected the oil extraction yield. Box–Behnken design (BBD) and response surface methodology (RSM) were used to optimize the extraction process. The optimal operating parameters were: 24 g/L NaCl, 300 W microwave power, 4:1 water to material ratio, an 80 min extraction time, and 45 °C extraction temperature. The chemical composition of the extracted seed oil was analyzed using gas chromatography–mass spectrometry (GC-MS). This extraction technique for yellow horn seed oil provided high throughput and high-quality oil. The present research offers a kind of green extraction method for edible oil in the food industry.

## 1. Introduction

Yellow horn (*Xanthoceras sorbifolium*) is a woody perennial shrub in the soapberry family, Sapindaceae. It is native to northwestern China, where it is well-adapted to cold, drought, salt, and starvation [1]. Yellow horn can live well below −40 °C except on saline–alkali soils or waterlogged fields. It is an important oil crop in China because of its abundant seed oil content (55–65%) [2]. The high content of linoleic acid is favorable for medicinal and nutritional application due to its cardioprotective, antidiabetic, and antimicrobial properties [3]. In addition, the kernel contains nutritionally valuable substances such as carbohydrates, fats, proteins, steroids, terpenoids, coumarins, flavonoids, organic acids, anthraquinones, and other compounds [4].

There are several industrial extraction methods (Table 1) for yellow horn oil including the expeller pressing method, solvent extraction, supercrital extraction, and water generation method. These methods have limited application because the setup costs are high, and the use of organic solvents contaminates the environment and is harmful to human health [2,5]. Furthermore, the defatted oilseed kernels, which contain nutritional and healthy constituents, have to be discarded as useless residues after oil extraction. For these reasons, a green and economical extraction method is needed [1]. An aqueous salt process (ASP) is a simple and green demulsification technology of water-in-oil emulsions. The demulsification efficiency may reach 100% in a very short time under microwave radiation. Microwave assisted extraction (MAE) is an accepted alternative to conventional extraction techniques, whereby microwave irradiation generates an electromagnetic field to accelerate the movement of molecules during the extraction process [6]. A methodology that combines these two techniques (ASP–MAE) may provide a viable alternative to the current methods.

Demand for efficient and green oil extraction techniques has increased [7], so the potential for using microwave and salt effects in the aqueous extraction in this research is just meeting the demand. The objective of this research is to optimize MAE under varying salt conditions of seed oil, which might provide valuable data for the economic process, green design, and pilot-scale, and the salt effects will also be evaluated.

This study aims to develop an ASP-MAE method for extracting oil from yellow horn seeds. The main factors affecting MAE efficiency are temperature, duration, and solvent [8]. Hence, our extraction variables included extraction temperature, the water to material ratio, and the extraction time. Response surface methodology (RSM) is an ideal tool for process optimization. Hence, we used it to optimize the extraction process. Furthermore, we analyzed the fatty acid composition to investigate the quality of the extracted seed oil using gas chromatography–mass spectrometry (GC-MS).

## 2. Results and Discussion

### 2.1. Optimisation of ASP-MAE

#### 2.1.1. Effect of Single Factors on Oil Yield

**Figure 1:**
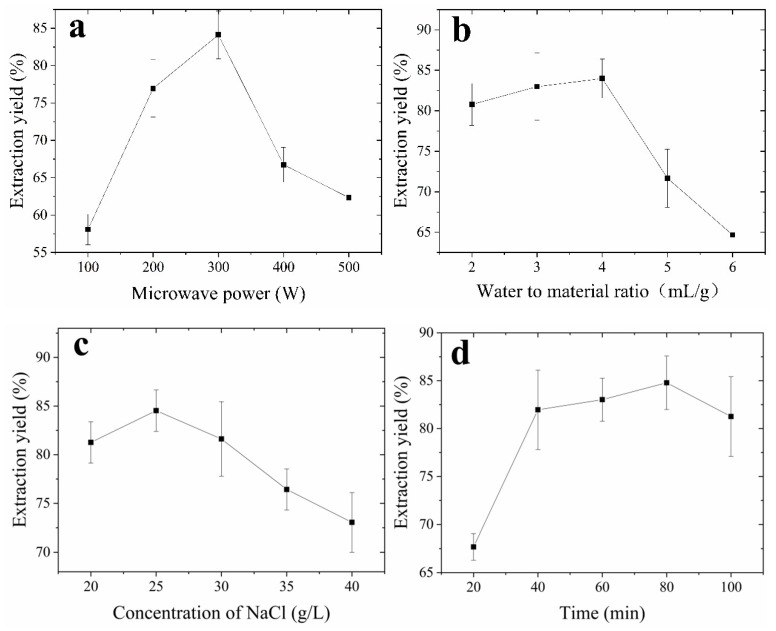

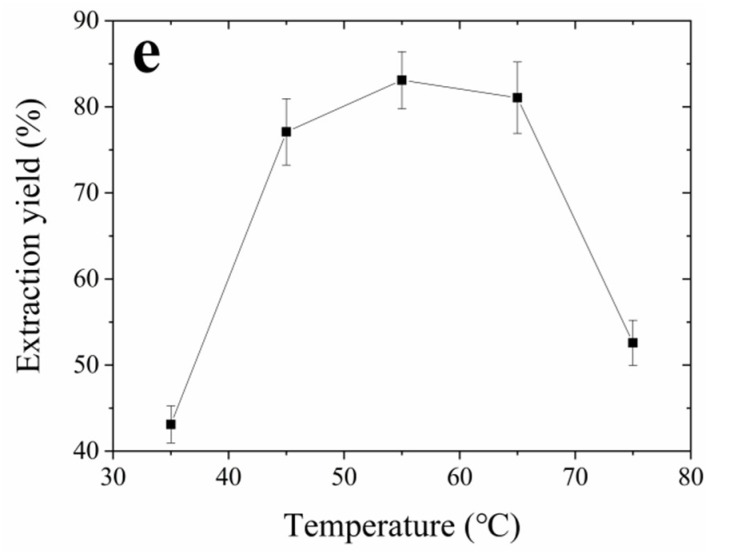
The effect of microwave power, water to material ratio, NaCl concentration, extraction time, and extraction temperature on oil extraction. (**a**) water to material ratio of 1:4 (w/v), 25 g/L NaCl, 60 min extraction at 45 °C; (**b**) 300 W microwave, 25 g/L NaCl, 60 min extraction at 45 °C; (**c**) 300 W microwave, water to material ratio of 1:4 (w/v), 60 min extraction at 45 °C; (**d**) 300 W microwave, water to material ratio of 1:4 (w/v), 25 g/L NaCl, 45 °C extraction; (**e**) 300 W microwave, water to material ratio of 1:4 (w/v), 25 g/L NaCl, 60 min extraction. Data are means ± SD (n = 3).

Materials were extracted by MAE at 100–500 W and oil was separated, meanwhile the other invariant extraction parameters were the water to material ratio of 4:1 (v/w), NaCl concentration of 25 g/L, and extraction duration of 60 min. The extraction yield of oil was positively correlated with increasing irradiation power, reaching 84.11 ± 3.21% at 300 W (Figure 1a).

The ratio of water to material is an important factor that can influence the extraction efficiency. The effect of the ratio of water to material on oil yield is shown in Figure 1b. The ratios of water to material were set at 2:1, 3:1, 4:1, 5:1, 6:1 (v/w), with the other extraction parameters constant: 300 W microwave power, 25 g/L NaCl, 45 °C extraction for 60 min. Oil yields increased with increasing ratio of water to material. The extraction yield reached its peak value (83.97 ± 2.41%) when the ratio of water to material was 4:1. When the ratios of water to material were higher than 4:1, the oil extraction yield decrease gradually, which may be due to the decrease of oil–water separation effect caused by the high ratio of water to material.

The concentration of NaCl is an important factor that can influence the extraction efficiency. The effect of NaCl concentration on oil yield is shown in Figure 1c. NaCl concentrations were set at 20, 25, 30, 35, and 40 g/L with the other extraction parameters constant: 300 W microwave power, 4:1 (v/w) ratio of water to material, 45 °C extraction for 60 min. The extraction yield of oil was positively correlated with increasing NaCl concentration. The maximum yield reached 84.53 ± 2.13% with 25 g/L NaCl. This is mainly due to the presence of inorganic salts; on the one hand break down of the electric double layer of emulsion interface increased the density difference between oil and water two phase; and on the other hand, inorganic salt increased the polarity difference of the oil and water, the solubility of the oil in water is reduced, enhancing salt-assisted effect for demulsification under microwave radiation [9].

The effect of extraction time on the yield of oil was studied with the extraction power of 300 W, and the other conditions were fixed at a water to material ratio of 4:1 (v/w) and extraction temperature 45 °C. Extraction times from 20 to 100 min had a positive linear effect on the oil yield (Figure 1d). Longer extraction times may have induced oil degradation, which will reduce yields.

The effect of extraction temperature on oil yields was studied with the extraction power of 300 W, and the other conditions were fixed at a water to material ratio of 4:1 (v/w), and extraction time 80 min. The extraction temperature had a significant linear effect on the oil yield from 35 to 55 °C, with a maximum yield (83.10 ± 3.305%) at 55 °C; after this point, oil yield started to decline (Figure 1e).

#### 2.1.2. Statistical Analysis and Model Fitting

To highlight the most influential factors and possible interactions in this study, a BBD model was designed. Thus, the ratio of material to solvent, microwave power, and extraction time and temperature were included in the model. There were 29 permutations of the four individual parameters that entered into the BBD, according to the factorial design shown in Table 2.

Table 3 depicts the ANOVA data for the fitted model. The *P*-value of <0.0001 indicates that the model was significant and the lack-of-fit 0.2523 showed was not significant. The ANOVA for oil extraction yields produced a correlation coefficient (R^2^) of 0.9724 with the calculated model, which is in close agreement with the experimental results. In addition, a lack-of-fit statistics was used to test the adequacy of the model, high significant levels for these (*p* < 0.001) were obtained by statistical analysis. The results suggest that the model can well explain for the prediction of oil extraction from this method. The response and test variables are related according to the following second-order polynomial equation:

(1) $$\begin{array}{ll} Y= & 86.37+0.27X_{1}+1.07X_{2}-1.79X_{3}+0.13X_{4}-1.51X_{1}X_{2}+0.69X_{1}X_{3}+1.93X_{1}X_{4}\\ & +1.51X_{2}X_{3}+1.66X_{2}X_{4}+0.94X_{3}X_{4}-8.50X_{1}{}^{2}-4.63X_{2}{}^{2}-5.91X_{3}{}^{2}-7.83X_{4}{}^{2} \end{array}$$

where X_1_ is microwave power (W), X_2_ is the water to material ratio (ml/g), X_3_ is NaCl (%), X_4_ is time (min), and *Y* is yield of extraction (%).

#### 2.1.3. Response Surface Analysis

**Figure 2:**
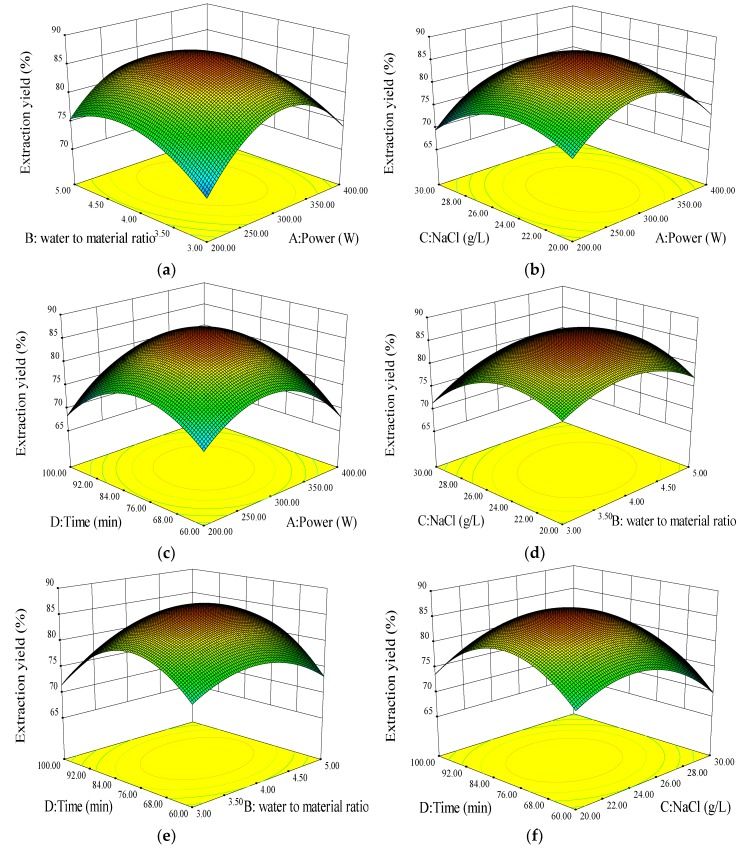
Tri-dimensional response surface showing the experimental factors and their mutual interactions on oil extraction. (**a**) microwave power and ratio of water to material, (**b**) microwave power and NaCl concentration, (**c**) microwave power and time, (**d**) ratio of water to material and NaCl concentration, (**e**) ratio of water to material and time, and (**f**) NaCl concentration and time.

To investigate the interactive effects of operational parameters on the yield of oil extraction, the three-dimensional profiles of multiple non-linear regression models and the two-dimensional contour generated by the model are illustrated in Figure 2. Two variables are depicted in a 3D surface plot while the other two variables are kept constant at zero level. The shapes of the contour plots, circular or elliptical, indicate whether mutual interactions between the variables are significant or not [10].

For oil yield, the interactions between microwave power (X_1_) and NaCl concentration (X_3_), and NaCl concentration (X_3_), and extraction time (X_4_) were not evident due to lack of or only weak functional relationship between the two variables in the break emulsification (Figure 2b,f).

Oil yield increased gradually with increasing microwave power and water to material ratio (Figure 2a) to a threshold level beyond which oil yield slightly decreased.

Figure 2c shows the response surface plot at various microwave powers and extraction times. Oil yield was higher at longer extraction times. However, the yield decreased with the increasing of extraction time. It indicated that the maximum extraction yield of oil could be achieved. This result indicated that extraction time had a different extent of influence on extraction yield in different microwave power.

Figure 2d shows the effects of water to material ratio and NaCl concentration on oil yield. As the water to material ratio and NaCl concentration increased, oil yields increased sharply from 20 to 25 g/mL NaCl but declined at higher concentrations.

Oil yield increased gradually with increasing water to material ratios and extraction times (Figure 2e) up to a threshold level, beyond which oil yield slightly decreased.

According to the RSM test results, the optional conditions of ASP–MAE process for oil extraction were 24 g/L NaCl, 300 W microwave power, 4:1 water to material ratio, and an 80 min extraction time. To validate the adequacy of the model equations, a verification experiment was carried out under the optimal conditions identified above. The model predicted a maximum response of 86.55%. The mean value of 84.62 ± 0.51% (n = 5) from the physical experiments verifies the validity of the extraction model. These findings confirm that the model is adequate for estimating the optimal combination of variables.

### 2.2. Antioxidant Activity

#### 2.2.1. Scavenging Activity of DPPH Radicals

**Figure 3:**
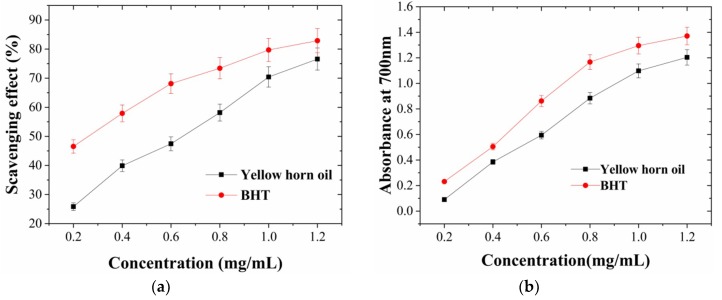
Activity of yellow horn oil and butylated hydroxytoluene (BHT) at different concentrations. (**a**) scavenging activity, (**b**) of reducing power activity. Data are means ± SD (n = 3).

The DPPH free-radical-scavenging effect of yellow horn oil is depicted in Figure 3a. It is an organic nitrogen radical with visible, ultraviolet absorption at 517 nm, and its color fades upon reduction [11]. As the concentration increased from 0.2 to 1.2 mg/mL, the DPPH radical-scavenging activity rose with increasing concentration of the extracted oils (Figure 3a). Beyond 1 mg/mL, the increasing became less obvious and the overall DPPH radical-scavenging activity was as strong as BHT. The results indicate that yellow horn oil had a noticeable effect on scavenging DPPH free radicals.

#### 2.2.2. Reducing Power Assay

In this assay, the ability of the sample to reduce Fe (III) to Fe (II) was determined and compared with BHT. The reducing power increased with increasing sample concentration (Figure 3b). The yellow horn oil showed higher reducing ability (absorbance of 0.4 at 700 nm) that was similar with the BHT. The reducing capacity of a compound may serve as a significant indicator of its potential antioxidant activity.

Studies have revealed that the potential antioxidant function of plant oils is mainly played by polyunsaturated fatty acids (PUFAs), tocopherols, and other components [12]. The relative contents of PUFAs (9,12-octadecadienoic acid) in the yellow horn oil extracted by ASP–MAE was 47.35% ± 3.79% in this study (Table 4). Moreover, current literature [13] indicates that yellow horn oil contains α-, γ-, and δ-tocopherols, of which γ-tocopherol is the major tocopherol, and the total tocopherol content is 83.28–106.27 mg/100 g for various extraction methods. The antioxidant activity of tocopherols is mainly attributed to their ability to donate hydrogen atoms to free radicals, thus inhibiting lipid oxidation.

### 2.3. Chemical Composition of the Extracted Seed Oil

The fatty acid profiles extracted from yellow horn oil via ASP-MAE or SE were analyzed using GC-MS (Table 4), with no differences observed between the extraction methods. The contents of monounsaturated fatty acids and polyunsaturated fatty acids in seed oil by ASP-MAE were slightly higher [14]. We conclude that yellow horn oil obtained by ASP-MAE is of excellent quality.

## 3. Experimental

### 3.1. Materials and Chemicals

Seeds of yellow horn were collected in the summer of 2017 from Gansu province, China, and identified by Prof. Xuelin Chen, College of Life Science, Northwest Normal University, Lanzhou, China. The seed capsules were manually cracked to release the seeds. The collected seeds were milled in a pulping machine (Langong 110, Kaifeng, China) prior to oil extraction. 

Sodium chloride of analytical grade was purchased from Fuchen Chemical Reagents Factory (Tianjin, China). 1,1-diphenyl-1-picrylhydrazyl (DPPH), trichloroacetic acid, and butylated hydroxytoluene (BHT) were purchased from Sigma-Aldrich (St. Louis, MO, USA). All other reagents were of analytical or HPLC grades.

### 3.2. Oil Extraction by ASP-MAE Method

Microwave extraction under varying salt levels was carried out using a microwave device with power settings ranging from 100 to 800 W (NJC 03-2, 2450 MHz, Nanjing Jiequan microwave equipment Co. Ltd., Nanjing, China). The microwave was equipped with a power sensor, a temperature sensor, a temperature controller and cooling system, and a special two necks round-bottomed flask. The amount of NaCl required for a certain concentration was added to ultra-pure water followed by 20 g of seed pulp and extraction solvent of a specified volume in an extraction flask. The MAE device was set to the preliminary conditions for extraction temperature, microwave power, and extraction time. After the scheduled time, the mixture obtained was transferred to a centrifuge tube and centrifuged for 10 min at 9000 rpm. The upper oil phase was collected and the other was used for subsequent research. The amount of extracted oil was gravimetrically analyzed, and the yield expressed as the percent ratio of the mass of extracted oil to the mass of oil for Soxhlet extraction is as follows:

(2) $$\mathrm{Extraction}\;\mathrm{yield}\;\mathrm{of}\;\mathrm{seed}\;\mathrm{oil}(\%)=(\frac{\mathrm{mass}\;\mathrm{of}\;\mathrm{extracted}\;\mathrm{oil}(g)}{\mathrm{mass}\;\mathrm{of}\;\mathrm{extracted}\;\mathrm{oil}\;\mathrm{for}\;\mathrm{Soxhlet}\;\mathrm{extraction}(g)\;})\times 100\%$$

### 3.3. Soxhlet Extraction of Seed Oil

An optimized Soxhlet extraction method (SE) was performed for the comparison with the ASP–MAE extraction [15]. Twenty grams of milled yellow horn seed kernels were extracted with petroleum ether (60–90 °C) in a Soxhlet extractor by heat reflux at 75 °C for 10 h. The extract was filtered, and petroleum ether in the filtrate removed at 40 °C under reduced pressure using a rotary evaporator. The extracted oil was weighed to calculate the extraction yield.

### 3.4. Box-Behnken Design (BBD)

Box–Behnken statistical design was used to statistically optimize the parameters of extraction conditions and to evaluate the main effects [16], interaction effects, and quadratic effects of the influencing factors on the seed oil yield (Table 5). The BBD identified strong effects of microwave power (W), the water to material ratio (mL/g), NaCl concentration, and extraction time on oil yield, and were used as the tested variables in a 29-group experiment. As shown in Table 1, the four factors selected for this study were designated as X_1_, X_2_, X_3_, and X_4_ and prescribed three levels, coded 1, 0, and −1 for high, intermediate, and low value, respectively. All experiments were performed in triplicate, with the averages for seed oil yield taken as a response. To predict the optimal point, a second-order polynomial model was fitted to correlate the relationship between independent variables and response. Test variables were coded according to the following equation:

(3) $$x_{i}=\frac{X_{i}-X_{0}}{\Delta X}$$

where *x_i_* is the coded value of an independent variable; *X_i_* is the actual value of an independent variable; *X_0_* is the actual value of an independent variable at the center point; △*X* is the step change value of an independent variable. For the three factors, the equation is:

(4) $$Y=A_{0}+\sum A_{i}X_{i}+\sum_{}^{}A_{ii}X_{i}+\sum_{}^{}A_{ij}X_{i}Y_{j}$$

where *Y* is the response variable (yield of seed oil in real values); *A_0_*, *A_i_*, *A_ii_*, *A_ij_* are the regression coefficients of variables for intercept, linear, quadratic, and interaction terms respectively; and *X_i_* and *X_j_* are the independent variables (*i* ≠ *j*). The variables of each factor were transferred to a scale between −1 and 1 for the appraisals, while the dependent variable was the oil extraction yield. According to the analysis of variance, the effect and regression coefficients of individual linear, quadratic, and interaction terms were determined. The regression coefficients were then used to make a statistical calculation to generate dimensional and contour maps from the regression models.

### 3.5. Evaluation of Physicochemical Properties

#### 3.5.1. Gas Chromatography-Mass Spectrometry (GS-MS) Analysis

The reference standard for the preparation of fatty acid methyl esters was the ester exchange method which is part of the Chinese national standard GB/T17376-2008.

A GC-MS analysis was performed using a gas chromatography/mass spectrometer (Thermo electron, Milan, Italy) equipped with an HP-5 silica capillary column (30 m × 0.25 mm × 0.25 μm, model HP6820, Hewlett-Packard, Palo Alto, CA, USA). The column temperature was initially set to 160 °C (held for 3 min), then increased to 210 °C at 2 °C/min (held for 1 min) and to 250 °C at 5°C/min (held for 1 min). The mass spectrometer was operated in positive ion mode with ionization energy of 70 eV. Injector and detector temperatures and the ion source temperature were 250 °C. Helium was used as a carrier gas, and the split ratio was 50:1. The retention indices and mass spectra, provided by the GC-MS controlling system, of the oil components were compared with the database of National Institute of Standards and Technology (NIST, 3.0).

#### 3.5.2. DPPH Radical-Scavenging Assay

The DPPH radical-scavenging effect of the extracts and essential oil was estimated using the method described by Brand-Williams, et al. [17] with some modifications. Briefly, 0.1 mL of extract or essential oil solution was mixed with 2 mL of DPPH solution with an absorbance at 517 nm. The mixture was incubated for 30 min at 23 °C. The absorbance was then measured at 517 nm. BHT was used as the reference compound. The DPPH radical-scavenging activity (%) was calculated from the following equation:

(5) $$\mathrm{Scavenging}\;\mathrm{effect}\;(\%)=\frac{A_{0}-\left(A_{s}-A_{x}\right)}{A_{0}}\times 100\%$$

where A_0_ is the absorbance of DPPH solution without a sample, A_s_ is the absorbance of the test sample mixed with DPPH solution, and A_x_ is the absorbance of the sample without DPPH solution.

#### 3.5.3. Reducing Power Assay

The reducing power of the yellow horn oil samples was determined using the method of Zeng et al. [18]. Accordingly, 1 mL of yellow horn oil sample (2–20 mg/mL) was mixed with 2.5 mL of phosphate buffer (0.2 M, pH 6.6) and 2.5 mL of potassium ferric cyanide solution (1%). The resulting mixture was incubated at 50 °C for 20 min and then cooled rapidly. To this mixture, 2.5 mL of trichloroacetic acid solution (10%) was added, mixed well, and then centrifuged for 10 min at 3000 rpm. The upper layer of the solution (2.5 mL) was diluted with distilled water (2.5 mL), and 0.5 mL of ferric chloride solution (0.1%) was added and mixed. The absorbance of the mixture was measured at 700 nm. A higher absorbance indicated a higher reducing power. BHT was used as the reference compound.

### 3.6. Statistical Analysis

The SPSS 17.0 software package was used to analyze the experimental data. *P*-values of less than 0.05 were considered statistically significant. All statistical analyses were performed with Origin 8.0 (Microcal Software Inc., Northampton, MA, USA). 

## 4. Conclusions

The study identified that ASP–MAE is an efficient, environmentally friendly, and easy procedure for oil extraction, and the optional parameters of the extraction process were 24 g/L NaCl, 300 W microwave power, 4:1 water to material ratio, and an 80 min extraction time; high-quality oil from yellow horn can be obtained under these extraction conditions. The antioxidant showed that yellow horn oil had a noticeable effect on scavenging DPPH free radicals and reducing capacity indicator of its potential antioxidant activity.

In all, the analyzed results of antioxidant activities and chemical composition demonstrate that yellow horn oil obtained from this emerging method can be used as a high-quality edible oil for the food industry in the future.

## Figures and Tables

**Table 1:** Major differences between the four extraction methods for yellow horn seed oil.

Oil Extraction Method	Oil Yield	Time (h)	Temperature (°C)	Cost
Expeller pressing method	57.25%	1–2	room temperature	Low
Solvent extraction	60.43%	5–10	70	High
Supercrital extraction	56.47%	2.5–3	50	High
Water generation method	58.74%	1–2	40–60	Low

**Table 2:** Operating parameters and the experimental and predicted values for oil extraction yields for the different experimental treatments.

Run	Microwave Power (W)	Water to Material Ratio	NaCl (g/L)	Time (min)	Extraction Yield (%)	Predicted Yield (%)
1	300	3:1	25	60	73.67 ± 2.77	74.36
2	300	3:1	30	80	71.04 ± 3.02	71.46
3	300	4:1	25	80	86.73 ± 2.89	86.37
4	200	4:1	30	80	71.34 ± 3.45	69.21
5	300	4:1	30	60	69.64 ± 3.30	69.77
6	200	3:1	25	80	69.67 ± 3.19	70.39
7	300	4:1	20	100	73.17 ± 2.67	73.60
8	400	4:1	25	60	68.64 ± 2.83	68.25
9	300	5:1	25	100	77.89 ± 2.92	76.76
10	300	4:1	25	80	85.88 ± 3.25	86.37
11	300	4:1	30	100	71.77 ± 3.33	71.90
12	300	4:1	25	80	86.54 ± 3.74	86.37
13	300	3:1	25	100	70.71 ± 3.56	71.31
14	200	5:1	25	80	73.47 ± 3.38	75.55
15	200	4:1	25	60	71.41 ± 2.83	71.57
16	300	5:1	20	80	77.73 ± 2.80	77.18
17	400	4:1	30	80	70.48 ± 3.43	71.13
18	300	4:1	25	80	84.97 ± 4.09	86.37
19	400	5:1	25	80	73.23±2.65	73.07
20	300	4:1	20	60	74.79 ± 1.91	75.23
21	300	3:1	20	80	78.98 ± 3.42	78.05
22	300	5:1	25	60	74.23 ± 2.36	73.20
23	400	4:1	25	100	72.65 ± 3.02	72.36
24	300	4:1	25	80	87.75 ± 1.64	86.37
25	400	3:1	25	80	75.45 ± 2.37	73.94
26	200	4:1	20	80	75.25 ± 1.29	74.17
27	200	4:1	25	100	67.71 ± 1.06	67.97
28	300	5:1	30	80	75.82 ± 2.99	76.62
29	400	4:1	20	80	71.63 ± 1.78	73.32

**Table 3:** ANOVA of quadratic model for the compositions of yellow horn oil.

Source	Sum of Squares	df	Mean Square	F-Value	*P*-Value Prob > F	
Model	925.6534	14	66.1181	35.1998	<0.0001	Significant
X_1_	0.8694	1	0.8694	0.4629	0.5074	
X_2_	13.7602	1	13.7602	7.3256	0.0170	
X_3_	38.3776	1	38.3776	20.4314	0.0005	
X_4_	0.1925	1	0.1925	0.1025	0.7536	
X_1_ X_2_	9.0601	1	9.0601	4.8234	0.0454	
X_1_ X_3_	1.9044	1	1.9044	1.0139	0.3311	
X_1_ X_4_	14.8610	1	14.8610	7.9117	0.0138	
X_2_ X_3_	9.0902	1	9.0902	4.8394	0.0451	
X_2_ X_4_	10.9561	1	10.9561	5.8328	0.0300	
X_3_ X_4_	3.5156	1	3.5156	1.8716	0.1928	
X_1_^2^	468.9611	1	468.9611	249.6642	<0.0001	
X_2_^2^	139.2204	1	139.2204	74.1178	<0.0001	
X_3_^2^	226.8738	1	226.8738	120.7824	<0.0001	
X_4_^2^	398.0942	1	398.0942	211.9363	<0.0001	
Residual	26.2971	14	1.8784			
Lack of Fit	22.0342	10	2.2034	2.0675	0.2523	Not significant
Pure Error	4.2629	4	1.0657			
Cor Total	951.9505	28				

**Table 4:** Fatty acid profiles and relative contents of yellow horn oil by ASP–MAE and SE.

No.	Component	Molecular Formula	Relative Content (%)	
			ASP–MAE	SE
1	Hexadecanoic acid	C_16_H_32_O_2_	3.40 ± 0.26	4.41 ± 0.37
2	9,12-Octadecadienoic acid	C_18_H_32_O_2_	47.35 ± 3.79	45.02 ± 4.05
3	9-Octadecenoic acid	C_18_H_34_O_2_	27.25 ± 2.13	30.02 ± 3.71
4	Octadecanoic acid	C_18_H_36_O_2_	1.51 ± 0.11	1.63 ± 0.15
5	15-Tetracosenoic acid	C_24_H_46_O_2_	1.99 ± 0.17	1.72 ± 0.13
6	11-Eicosenoic acid	C_20_H_38_O_2_	6.82 ± 0.76	6.31 ± 0.84
7	13-Docosenoic acid	C_22_H_42_O_2_	11.23 ± 0.87	10.15 ± 1.27
8	Docosanoic acid	C_22_H_44_O_2_	0.46 ± 0.04	0.73 ± 0.06

**Table 5:** Independent variables and their levels used in the response surface design.

Independent Variables	Symbol		Factor Level		
	Coded	Uncoded	−1	0	1
Microwave power (W)	x_1_	X_1_	200	300	400
Water to material ratio (mL/g)	x_2_	X_2_	3:1	4:1	5:1
NaCl (g/L)	x_3_	X_3_	20	25	30
Time (min)	x_4_	X_4_	60	80	100
